# Thymic hyperplasia with lymphoepithelial sialadenitis (LESA)-like features: a case report and literature review

**DOI:** 10.1186/s13000-023-01391-z

**Published:** 2023-09-11

**Authors:** Wenfeng Xu, Long Wang, Hao Tang, Ling Luo, Yujuan Xu, Deyu Guo

**Affiliations:** 1 Department of Pathology, Guiqian International General Hospital, Guiyang City, Guizhou Province China; 2 Department of Radiology, Guiqian International General Hospital, Guiyang City, Guizhou Province China

**Keywords:** Thymic hyperplasia, Thymic hyperplasia with lymphoepithelial sialadenitis-like features, Pathology, Lymphoma

## Abstract

**Background:**

Thymic hyperplasia with lymphoepithelial sialadenitis-like features (LESA-like TH) is a rare form of thymic hyperplasia, characterized by a prominent expansion of the thymic medulla containing hyperplastic lymphoid follicles with germinal centers, while an almost total absence of thymic cortex. Since the first report in 2012, only a few cases of LESA-like TH have been reported in the literature to date. Due to the rarity of LESA-like TH and the tumor-like morphology, it is easy to be misdiagnosed as other common diseases of the thymus in routine practice, such as thymoma and lymphoma.

**Case presentation:**

Herein, we present a case report of a 52-year-old Chinese female patient with LESA-like TH, without any discomforting symptoms. Computer-tomography imaging revealed a cystic solid mass in the anterior mediastinum, with well-defined boundaries and multiple internal septa. Histologically, prominent features were florid lymphoid follicles containing germinal centers, as well as hyperplasia of thymic epithelial cells and proliferation of Hassall bodies. However, the thymic cortex rich in immature T cells was almost completely absent. Furthermore, mature plasma cells, lymphoepithelial lesions, and cholesterol clefts were frequently seen.

**Conclusion:**

We made a diagnosis of LESA-like TH and performed a literature review to better understand the clinicopathological features of LESA-like TH and reduce misdiagnosis.

## Introduction

Thymic hyperplasia with lymphoepithelial sialadenitis-like features (LESA-Like TH) is a rare nonneoplastic but tumor-like thymic hyperplasia, characterized by massive lymphoid follicles with germinal centers, hyperplasia of the thymic epithelium and proliferation of the Hassall bodies, scattered lymphoepithelial lesions, and focal cystic changes. This disease was first reported in 2012 by Weissferdt et al. [1]. Since then, merely a total of 45 cases documented in 3 literatures in English, 1 literature in German, and 1 literature in Chinese have been reported to date [1–5]. The rare incidence of LESA-Like TH leads to a limited understanding of its clinicopathological features, and its tumor-like morphology makes it easy to be misdiagnosed as other common diseases of the thymus in routine practice, such as thymoma and lymphoma. However, surgical resection is the ideal treatment modality for LESA-like TH and the prognosis is favorable [1, 2, 5]. Therefore, the correct pathological diagnosis is of great significance for the clinical management of patients. Herein, we presented a case of LESA-like TH, and performed a literature review in order to better understand the clinicopathological features of LESA-like TH and reduce misdiagnosis.

## Case presentation

**Fig. 1:**
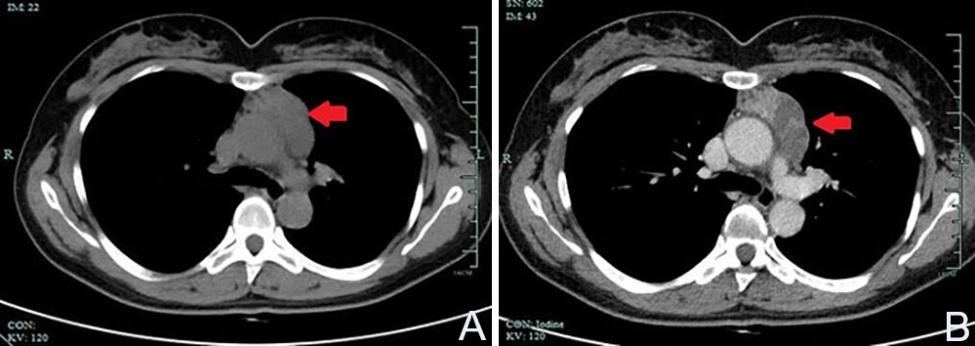
Chest-computed tomography revealed a cystic solid mass in the anterior mediastinum (**A**). Well-defined boundaries and mild to moderate heterogeneous enhancement of the solid portion and internal septum were detected by computed tomography-enhanced scans (**B**)

A 52-year-old female patient was admitted to our hospital due to a mediastinal mass found fortuitously for a month. Chest-computed tomography showed a cystic solid mass measuring 25 × 51 × 60 mm in the anterior mediastinum, with multiple septa inside. Computed tomography-enhanced scans further revealed well-defined boundaries and mild to moderate heterogeneous enhancement of the solid portion and internal septum (Fig. 1). However, the patient was asymptomatic, previously healthy, and had no history of smoking or drinking. Laboratory examination showed no abnormalities, except for a mild increase in the carbohydrate antigen 724, a tumor marker. Then, total surgical resection and pathological biopsy were performed on the fifth day after admission to confirm the diagnosis.

## Pathological findings

**Fig. 2:**
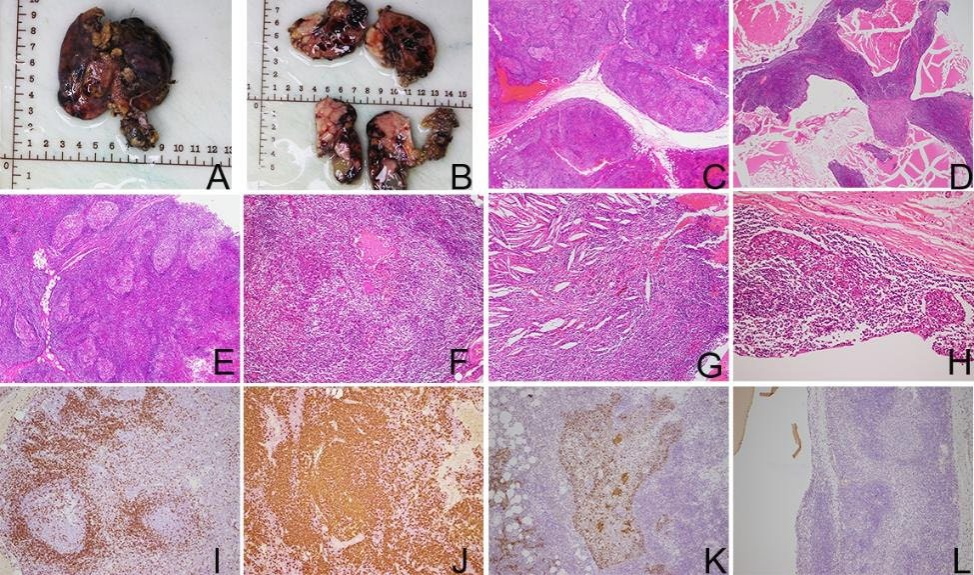
(**A**). Grossly, the anterior mediastinal mass resembled an enlarged thymus gland; (**B**). the cut surfaces were gray-white in color and medium in texture, showing multinodular architecture and focal cystic changes; (**C**). on the low magnification (H&E, ×20), predominantly nodular or lobulated solid structures separated by fibrous and adipose tissue; (**D**). focal cystic structures (H&E, ×20); (**E**). on the higher magnification, the lobulated solid structures were characterized by prominent lymphoid follicles containing germinal centers and florid hyperplasia of thymic epithelial cells (H&E, ×40); (**F**). proliferation of dilated Hassall bodies (H&E, ×100); (**G**). cholesterol clefts (H&E, ×100); (**H**). lymphoepithelial lesions (H&E, ×200); (**I**). CD3 + T cells scattered in the interfollicular areas (EnVision, ×100); (**J**). CD20 + B cells mainly populated in lymphoid follicles (EnVision, ×100). (**K**). pan-cytokeratin staining showed reticular hyperplasia of thymic epithelial cells (EnVision, ×100); (**L**). TDT + immature T cells were almost completely absent (EnVision, ×100)

Grossly, the intact resection specimen resembled a lobulated thymus gland but was obviously enlarged in size beyond normal limits, measuring 75 × 70 × 15 mm. The cut surfaces were solid and focally cystic. Solid areas consisted of gray-white or gray-brown multi-nodular structures with medium texture. Cystic areas were composed of several cystic cavities containing gray-red translucent jelly-like substances, with diameters of 2–15 mm (Fig. 2A,B).

On low power, a pattern characterized by predominantly nodular or lobulated solid structures separated by fibrous and adipose tissue, and focally cystic structures was observed (Fig. 2C,D). On higher magnification, the most prominent features of solid areas were characterized by the florid proliferation of lymphoid follicles with germinal centers, thymic epithelium, and Hassall bodies. Clusters, nests, and reticulations of bland thymic epithelial cells were observed in the interfollicular areas or surrounding lymphoid follicles. In addition, lymphoepithelial lesions formed by bland thymic epithelial cells with lymphocytes, and scattered mature plasma cells were observed in the interfollicular areas. Focally, cholesterol clefts and their resulting cholesterol cleft granulomas were seen. Cysts were covered by squamous epithelium, without cytologic atypia, and contained homogeneous eosinophilic amorphous matter (Fig. 2E-H).

Immunohistochemical results showed that lymphoid follicles expressed B-cell marker CD20, with scattered CD3 + T cells and CD20 + B cells populated in the interfollicular areas, however, TDT + immature T cells were almost completely absent. Pan-cytokeratin marker clearly delineated hyperplastic, nested, or reticular thymic epithelial cells and lymphoepithelial lesions (Fig. 2I-L). CD38 + mature plasma cells were observed in the interfollicular areas, and kappa and Lambda staining confirmed the polyclonal process.

Finally, a diagnosis of lymphoepithelial sialadenitis-like thymic hyperplasia was made. The patient recovered well postoperatively, and at a follow-up examination 2 months after surgery, we found no evidence of discomforting symptoms and recurrence or metastasis (Table 1).

**Table 1:** A summary of 45 cases (including the current case) of LESA-like TH in the literatures

References	Number of cases	Gender(M/F)	Median age (range)(years)	Location	Median size (range)(mm)	Clinical Manifestations	Autoimmune disorders	Treatment	Outcome
Weissferdt et al. [1]	4	2/2	45.5(37–53)	Anterior mediastinum	229 (115–220)	3 cases asymptomatic,1 case chest pain	Not found	Surgery	Alive
ZHANG et al. [4]	2	1/1	61 (56–66)	The anterior middle and upper mediastinum	85 (60–130)	1 case asymptomatic,1 case cough and phlegm	Not found	Surgery	Alive
Tanaka et al. [2]	1	1/0	55	Anterior mediastinum	240	Asymptomatic	Immunoglobulin G4-related disorders	Surgery	Alive
Porubsky et al. [3]	36	21/15	52 (32–80)	Unknown	70 (10–145)	34 cases asymptomatic, 2 cases MG	12 patients autoimmune disorders	Unknown	Unknown
Arndt et al. [5]	1	1/0	43	Upper mediastinum	45	Dyspnea and chest pain	Not found	Surgery	Alive
Current case	1	0/1	52	Anterior mediastinum	75	Asymptomatic	Not found	Surgery	Alive

Abbreviations: M, Male; F, Female; MG, myasthenia gravis

## Discussion

In 2012, Weissferdt et al. first reported 4 patients with thymic hyperplasia similar to lymphoepithelial sialadenitis (LESA) of the salivary glands and named them “thymic hyperplasia with lymphoepithelial sialadenitis-like features” [1]. Currently, the disease is considered a rare form of thymic hyperplasia that commonly affects people in their fourth or fifth decades of life without a sex predilection [1–4]. However, the incidence rate is unclear. Patients with LESA-like TH usually appear an anterior mediastinal mass discovered incidentally on imaging studies. Most patients are asymptomatic, and a few patients suffer from a series of symptoms due to mass compression of adjacent tissues.

Macroscopically, the mass of LESA-like TH is enlarged both in size and weight. The cut surfaces are gray-white in color and medium in texture, showing multinodular architecture and focal cystic changes. Microscopically, its histological morphology shows a characteristic pattern of lobulated structures divided by fibrofatty tissue septum. There is abundant lymphofollicular hyperplasia with germinal centers, bland thymic epithelium with reticular or cord hyperplasia, and proliferation of dilated Hassall corpuscles. Mature plasma cells are scattered in the interfollicular areas, and polytypic light chain expression is confirmed by Kappa and Lambda immunohistochemical staining. In addition, the presence of lymphoepithelial lesions, cholesterol clefts and their resulting cholesterol cleft granulomas, and cystic structures lined by squamous epithelium can be observed. Immunohistochemically, epithelial markers such as pan-cytokeratin and CK19 can clearly delineate the obvious hyperplasia of thymic epithelial cells as well as Hassall corpuscles, and lymphoepithelial lesions in the medullary region. Staining for lymphocyte markers reveals a massive proliferation of mature B and T lymphocytes, while an almost complete absence of cortical areas rich in immature T cells is detected.

Morphologically, LESA-like TH striking resembles LESA of the salivary gland. LESA is a component of SjÖgren’s syndrome, characterized by lobulated structure, reactive lymphoid follicles with germinal centers, hyperplasia of ducts, and lymphoepithelial lesions [6]. It is reported that patients with LESA had a 44-fold increased risk of developing lymphoma, with mucosa-associated lymphoid tissue lymphoma (MALT lymphoma) being the most common [7–9]. Initially, Weissferdt et al. also suggested that LESA-like thymic hyperplasia might be a precursor lesion of thymic MALT lymphoma [1]. Until 2020, Porubsky et al. reported the largest cohort of patients with LESA-like TH and found that 10% of patients had concomitant lymphoma, especially MALT lymphoma, and 33% of patients were associated with non-myasthenic autoimmune diseases. Furthermore, residual LESA-like TH was found in a retrospective analysis of primary thymic MALT lymphoma. Therefore, Porubsky et al. suggested that LESA-like TH may be the precursor lesion of thymic MALT lymphoma as well [3]. However, more relevant case reports are warranted to confirm.

The primary differential diagnosis of LESA-like TH is thymic MALT lymphoma. The median age at onset of thymic MALT lymphoma is 63 years old, about a decade later than LESA-like TH [10]. Likewise, the presence of cystic changes, cholesterol granulomas, and lymphoid follicles can be observed in thymic MALT lymphoma, while thymic epithelium and Hassall corpuscles are occasionally seen. Most importantly, lymphoid follicles in thymic MALT lymphoma have prominent marginal zones, and lymphocytes are characterized by small atypical and monotonous sheets of lymphocytes. These heteromorphic lymphocytes percolate the residual epithelium to form lymphoepithelial lesions or destroy the germinal centers of lymphoid follicles—termed follicular “colonization”, which is absent in LESA-like TH [10]. It is known that thymic MALT lymphoma is a clonal B-cell proliferation lesion, while clonal B-cell proliferation can be detected in a few patients with LESA-like TH as well [3]. Therefore, it is important to underscore that the differential diagnosis from thymic MALT lymphoma cannot be based solely on molecular detection, but also needs to be combined with morphological features.

The thymus gland weighs about 25 g at birth and reaches a maximum weight of about 30-40 g at puberty, then begins to be gradually replaced by adipose tissue and degenerates, but does not disappear completely [11]. “Conventional” thymic hyperplasia mainly includes true thymic hyperplasia (TTH) and thymic follicular hyperplasia (TFH) [12]. TTH usually affects infants and children, and is defined as a remarkably enlarged both in size and weight above normal limits while preserving normal thymic architecture [13]. There are 2 types of TTH according to etiology and pathogenesis: (1) primary thymic hyperplasia without any other disease; (2) secondary thymic hyperplasia in association with other diseases including endocrine abnormalities, sarcoidosis, and Beckwith-Wiedemann syndrome [12]. On the other hand, TFH also shows abundant lymphoid follicles leading to expansion of the medullary region, but the normal architecture of the cortical region and clear corticomedullary boundary is preserved. Another notable feature that distinguishes it from LESA-like TH is the absence of apparent hyperplasia of the thymic epithelium and Hassall corpuscles. While cortical atrophy is sometimes seen in massive and long-standing TFH, there was almost complete absence of cortex in LESA-like TH [13]. In addition, TFH prefers younger female patients and is associated with myasthenia gravis [14].

Finally, LESA-like TH needs to be differentiated from the most common epithelial tumor of the thymus (thymoma) and benign cystic lesions of the thymus. Micronodular thymoma with lymphoid stroma (MNT) shares similar features with LESA-like TH, including cyst changes, lymphoid follicles, and the paucity of immature T cells. However, MNT shows islands of spindle-shaped neoplastic epithelium surrounded by lymphoid stroma free of epithelial cells [15]. Other subtypes of thymoma with lymphoid follicles were either associated with myasthenia gravis or enriched in TDT + immature T lymphocytes in corticosteroid naïve patients. In a similar pattern to LESA-like TH, multilocular thymic cysts (MTCs) are composed of multiple cysts lined by squamous or low cuboidal epithelium, and accompanied by inflammatory changes, lymphoid hyperplasia with germinal centers, the remnant of thymic tissue, cholesterol cleft granulomas, and dilated Hassall corpuscles. Although atypical epithelial pseudoepitheliomatous hyperplasia was reported in some MTCs, those changes are confined to the inner wall of cysts [16]. While nested or reticular hyperplasia of thymic epithelium, and lymphoepithelial lesions can be seen in the thymic parenchyma of patients with LESA-like TH.

In conclusion, we reported a Chinese patient with LESA-like TH and performed a literature review on the clinicopathological features of LESA-like TH. LESA-like TH is a rare thymic hyperplasia with tumor-like features. Due to its rare incidence, it is easy to be misdiagnosed in routine practice. Morphologically, it is characterized by a striking dilation of the thymic medullary area with lymphoid follicles containing germinal centers, hyperplastic thymic epithelial cells and Hassall bodies, lymphoepithelial lesions, plasma cell infiltration, and cystic changes, while the cortical area is mostly all absent. Therefore, LESA-like TH should be considered in the diagnosis of an anterior mediastinal mass characterized by hyperplasia of lymphoid follicular, hyperplasia of thymic epithelial cells, lymphoepithelial lesions, and the almost complete absence of the thymic cortex.

## Data Availability

All data are included in this article.

## Abbreviations

LESA-like TH: Thymic hyperplasia with lymphoepithelial sialadenitis-like features
LESA: Lymphoepithelial sialadenitis
MALT lymphoma: Mucosa-associated lymphoid tissue lymphoma
TTH: True thymic hyperplasia
TFH: Thymic follicular hyperplasia
MNT: Micronodular thymoma with lymphoid stroma
MTC: Multilocular thymic cyst

## References

[1] Weissferdt A, Moran CA (2012). Thymic hyperplasia with lymphoepithelial sialadenitis (LESA)-like features: a clinicopathologic and immunohistochemical study of 4 cases. Am J Clin Pathol.

[2] Tanaka N, Ishihara S, Inoue M, Konishi E. Thymic Hyperplasia with Lymphoepithelial Sialadenitis-Like features arising in a patient with immunoglobulin G4-Related Disorders: a Case Report [published online ahead of print, 2023 Jan 24]. Int J Surg Pathol. 2023;10668969221150530. 10.1177/10668969221150530. 10.1177/10668969221150530 36694416

[3] Porubsky S, Popovic ZV, Badve S et al. Thymic Hyperplasia with Lymphoepithelial Sialadenitis (LESA)-Like Features: Strong Association with Lymphomas and Non-Myasthenic Autoimmune Diseases. Cancers (Basel). 2021;13(2):315. Published 2021 Jan 16. 10.3390/cancers13020315. 10.3390/cancers13020315 PMC7830871 33467055

[4] Zhang XY, Mei KY, Wang H (2021). al.Thymic hyperplasia with lymphoepithelial sialadenitis-like features: a clinicopathologic and immunohistochemical analysis of two cases. J Diag Pathol.

[5] Arndt B, Gaiser T, Marx A, Rieger C (2016). Lymphoepitheliale Sialadenitis-artige Thymushyperplasie [Lymphoepithelial sialadenitis (LESA)-like thymic hyperplasia: a case report]. Dtsch Med Wochenschr.

[6] Ellis GL, Auclair PL (2007). Atlas of Tumor Pathology: tumors of the salivary glands.

[7] Falzon M, Isaacson PG (1991). The natural history of benign lymphoepithelial lesion of the salivary gland in which there is a monoclonal population of B cells. A report of two cases. Am J Surg Pathol.

[8] Kassan SS, Thomas TL, Moutsopoulos HM (1978). Increased risk of lymphoma in sicca syndrome. Ann Intern Med.

[9] Carbone A, Gloghini A, Ferlito A (2000). Pathological features of lymphoid proliferations of the salivary glands: lymphoepithelial sialadenitis versus low-grade B-cell lymphoma of the malt type. Ann Otol Rhinol Laryngol.

[10] Pina-Oviedo S (2021). Mediastinal Lymphoproliferative Disorders. Adv Anat Pathol.

[11] Ocal T, Türken A, Ciftçi AO, Senocak ME, Tanyel FC, Büyükpamukçu N (2000). Thymic enlargement in childhood. Turk J Pediatr.

[12] Deepali J, Justin AB, Mark RW (2020). Atlas of Thymic Pathology. Springer Nat Singap Pte Ltd.

[13] Weis CA, Märkl B, Schuster T, Vollert K, Ströbel P, Marx A (2017). „Echte Thymushyperplasie: Differenzialdiagnose der Thymusvergrößerung bei Säuglingen und Kindern [True thymic hyperplasia: Differential diagnosis of thymic mass lesions in neonates and children]. Pathologe.

[14] Mlika M, Ayadi-Kaddour A, Marghli A, Ismail O, Kilani T, El Mezni F (2009). True thymic hyperplasia versus follicular thymic hyperplasia: a retrospective analysis of 13 cases. Pathologica.

[15] Qu L, Xiong Y, Yao Q, Zhang B, Li T (2017). Micronodular thymoma with lymphoid stroma: two cases, one in a multilocular thymic cyst, and literature review. Thorac Cancer.

[16] Oramas DM, Moran CA (2022). Multilocular thymic cyst (MTC) and other tumors with MTC features: pitfalls in diagnosis. Semin Diagn Pathol.

